# A phase Ib/IIa, randomised, double-blind, multicentre trial to assess the safety and efficacy of expanded Cx611 allogeneic adipose-derived stem cells (eASCs) for the treatment of patients with community-acquired bacterial pneumonia admitted to the intensive care unit

**DOI:** 10.1186/s12890-020-01324-2

**Published:** 2020-11-25

**Authors:** Pierre-François Laterre, Miguel Sánchez-García, Tom van der Poll, Olga de la Rosa, Kathy-Ann Cadogan, Eleuterio Lombardo, Bruno François

**Affiliations:** 1 Intensive Care Unit, St Luc University Hospital, Université Catholique de Louvain, 10 avenue, 1200 Brussels, Belgium; 2 Intensive Care Department, Hospital Clínico San Carlos, Madrid, Spain; 3 The Center of Experimental and Molecular Medicine, Amsterdam University Medical Center, University of Amsterdam, Amsterdam, the Netherlands; 4 Takeda Madrid, Cell Therapy Technology Center, Tres Cantos, Spain; 5 Takeda Pharmaceuticals, Cambridge, MA USA; 6 Intensive Care Unit, and Inserm CIC1435 & UMR1092, Dupuytren University Hospital, Limoges, France

**Keywords:** Community acquired bacterial pneumonia, Mesenchymal stem cells, Adjunctive therapy, Clinical trial, Study protocol

## Abstract

**Background:**

Community-acquired bacterial pneumonia (CABP) can lead to sepsis and is associated with high mortality rates in patients presenting with shock and/or respiratory failure and who require mechanical ventilation and admission to intensive care units, thus reflecting the limited effectiveness of current therapy. Preclinical studies support the efficacy of expanded allogeneic adipose-derived mesenchymal stem cells (eASCs) in the treatment of sepsis. In this study, we aim to test the safety, tolerability and efficacy of eASCs as adjunctive therapy in patients with severe CABP (sCABP).

**Methods:**

In addition to standard of care according to local guidelines, we will administer eASCs (Cx611) or placebo intravenously as adjunctive therapy to patients with sCABP. Enrolment is planned for approximately 180 patients who will be randomised to treatment groups in a 1:1 ratio according to a pre-defined randomization list. An equal number of patients is planned for allocation to each group. Cx611 will be administered on Day 1 and on Day 3 at a dose of 160 million cells (2 million cells / mL, total volume 80 mL) through a 20–30 min (240 mL/hr) intravenous (IV) central line infusion after dilution with Ringer Lactate solution. Placebo (Ringer Lactate) will also be administered through a 20–30 min (240 mL/hr) IV central line infusion at the same quantity (total volume of 80 mL) and following the same schedule as the active treatment. The study was initiated in January 2017 and approved by competent authorities and ethics committees in Belgium, Spain, Lithuania, Italy, Norway and France; monitoring will be performed at regular intervals. Funding is from the European Union’s Horizon 2020 Research and Innovation Program.

**Discussion:**

SEPCELL is the first trial to assess the effects of eASCs in sCABP. The data generated will advance understanding of the mode of action of Cx611 and will provide evidence on the safety, tolerability and efficacy of Cx611 in patients with sCABP. These data will be critical for the design of future confirmatory clinical investigations and will assist in defining endpoints, key biomarkers of interest and sample size determination.

**Trial registration:**

NCT03158727, retrospectively registered on 9 May 2017.

**Supplementary Information:**

The online version contains supplementary material available at 10.1186/s12890-020-01324-2.

## Background

Community-acquired pneumonia (CAP) is an acute lung infection and the leading cause of mortality among infectious diseases [1]. CAP is a substantial public health issue, with an overall annual incidence ranging from 1.6–10.6/1000 adult population in Europe [2]. Community-acquired bacterial pneumonia (CABP), where CAP is triggered by bacterial pathogens such as *Streptococcus pneumoniae* and gram-negative organisms, is also a significant cause of disease complications, particularly sepsis [3]. Almost half (48%) of patients hospitalised due to CABP develop organ dysfunction during the disease course [4]. In severe CABP (sCABP), patients may experience respiratory failure, necessitating invasive mechanical ventilation, and/or hypotension, which may be refractory to intravascular volume expansion, thus requiring the use of vasopressors [3].

CAP is commonly caused by bacterial pathogens, and treated accordingly with antibiotic and supportive therapy [3]. However, despite advances in critical care management, the mortality rate of CAP has remained unacceptably high [5]. In cases where patients require intensive care unit (ICU) admission for additional care, mortality rates of approximately 25–50% have been reported [5]. Early treatment with appropriate antibiotics, usually a β-lactam plus a macrolide, may improve patient outcomes, particularly in those at a higher risk of death [3]. To date, the application of corticosteroid combination therapy in CAP/CABP has remained controversial, with some studies demonstrating reduced mortality [6–8], but others report a minimal influence on outcomes [9] and an increase in adverse effects [10]. Given the limited armamentarium for CAP management, there exists a clear unmet need for new therapeutic strategies to improve outcomes for patients with sCABP.

**Fig. 1:**
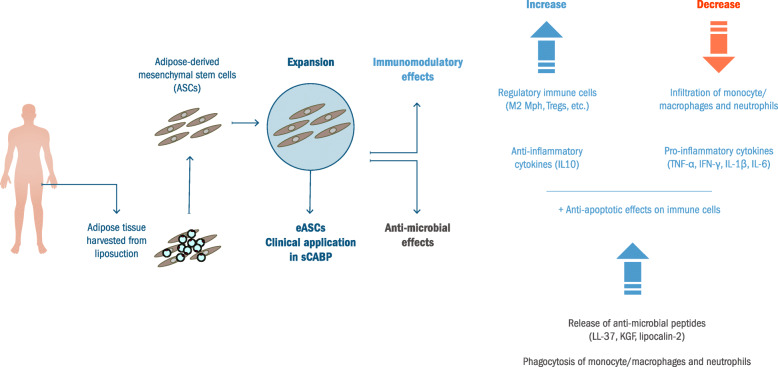
Adipose-derived mesenchymal stem cells are sampled from human adipose tissue and expanded ex vivo. Their immunomodulatory and anti-microbial effects are utilised for the treatment of sCABP. eASC MoA: eASCs modulate inflammation through the generation of regulatory immune cells (e.g. Tregs, M2 Mph) by reducing pro-inflammatory cytokines (e.g. TNFα, IL-6, IL-8); increasing the release of the anti-inflammatory cytokine IL-10; inhibiting apoptosis of immune cells; and reducing lymphocyte, neutrophil and macrophage infiltration. eASCs also have anti-microbial effects as they release peptides with antimicrobial properties (e.g. LL-37) and increase the phagocytic capacity of monocytes, macrophages and neutrophils. Due to these properties, eASCs can reduce organ injury and increase functionality, thus conferring a therapeutic benefit [11]. *eASC* expanded adipose-derived mesenchymal stem cell, *IFN* interferon, *IL* interleukin, *KGF* keratinocyte growth factor, *M2 Mph* macrophages, *MoA* mode of action, *sCABP* severe community-acquired bacterial pneumonia, *Treg* regulatory T cell, *TNFα* tumour necrosis factor alpha

Recently, several non-antibiotic therapies have been explored as adjuvant treatments, including neutralising antibodies against bacterial toxins, immunoglobulins, growth factors and mesenchymal stem cells (MSCs). MSCs have both immunomodulatory and anti-microbial effects, and possess the ability to modulate the phenotype and function of a range of immune cells through direct cell-to-cell interactions, immunomodulatory factors and secretion of growth factors, making them attractive candidates to treat diseases associated with a defective inflammatory response (Fig. 1) [11]. MSCs have received wide attention as a novel therapeutic candidate for various inflammatory medical conditions including graft versus host disease, consequences of myocardial infarction, and perianal fistula in Crohn’s disease [12–14], and several preclinical studies have examined the effectiveness of MSCs for the treatment of sepsis [15–21].

Although MSCs from different sources share many characteristics, significant variability in their immunomodulatory properties exist [22]. In contrast to bone-marrow-derived MSCs (BM-MSCs), adipose-derived mesenchymal stem cells (ASCs) can be obtained from human adipose tissue via liposuction and constitute an easily accessible source of stem cells [23]. Furthermore, ASCs display a reduced susceptibility to natural killer cell-mediated lysis compared to BM-MSCs, and may, therefore, remain in tissues long enough to balance the immune response before being cleared [24]. Additionally, compared with BM-MSCs, ASCs have been described as having greater immunomodulatory effects [25–29].

A previous phase I trial to investigate the safety and demonstrate the anti-inflammatory effect of expanded ASCs (eASCs) for treating sepsis-like clinical symptoms reported no serious adverse events (SAEs) nor respiratory complications in particular [19]. Here, we describe the study design and methodology for SEPCELL (NCT03158727), an ongoing phase Ib/IIa study to assess the safety, tolerability and efficacy of eASCs (Cx611) as adjunctive therapy in patients with sCABP.

### Objectives

The primary objective of SEPCELL is to determine the safety profile of two central line infusions of Cx611 administered within 3 days (on days 1 and 3) at a dose of 160 million cells each to monitor any AE and potential immunological host responses against the administered cells during the follow-up period. The secondary objectives are to explore the clinical efficacy of Cx611 in terms of a reduction of the duration of mechanical ventilation and/or the need for vasopressors and/or improved survival and/or clinical cure of the sCABP, as well as other efficacy-related endpoints. A further objective is to understand the mode of action (MoA) of Cx611 in patients with sCABP by identifying the pro-inflammatory and anti-inflammatory pathways through which Cx611 may affect the underlying processes of sepsis.

## Methods

### Design

**Fig. 2:**
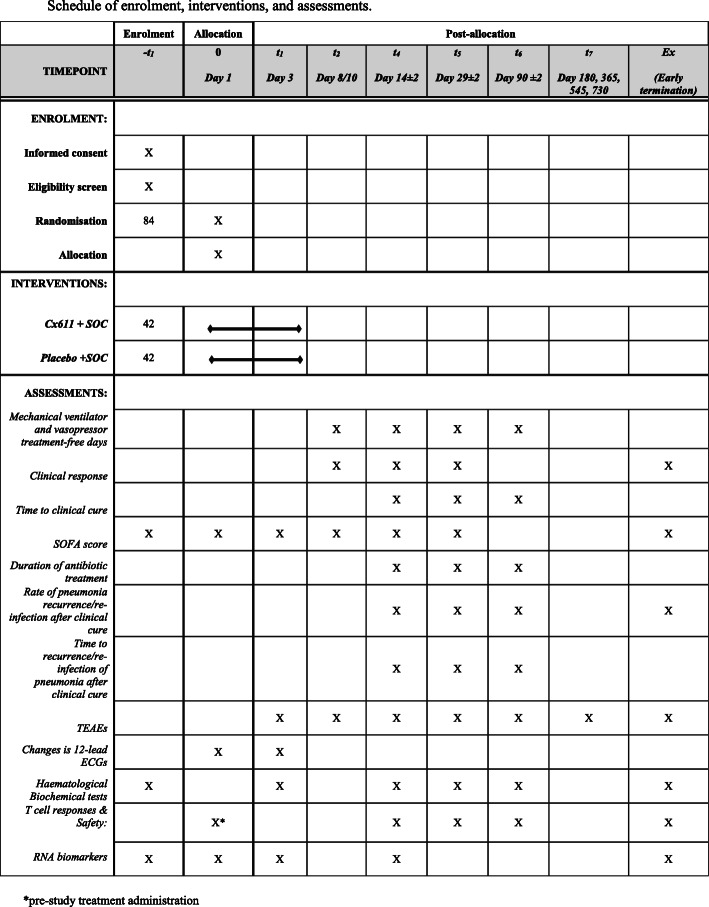
Schedule of enrolment, interventions, and assessments

**Fig. 3:**
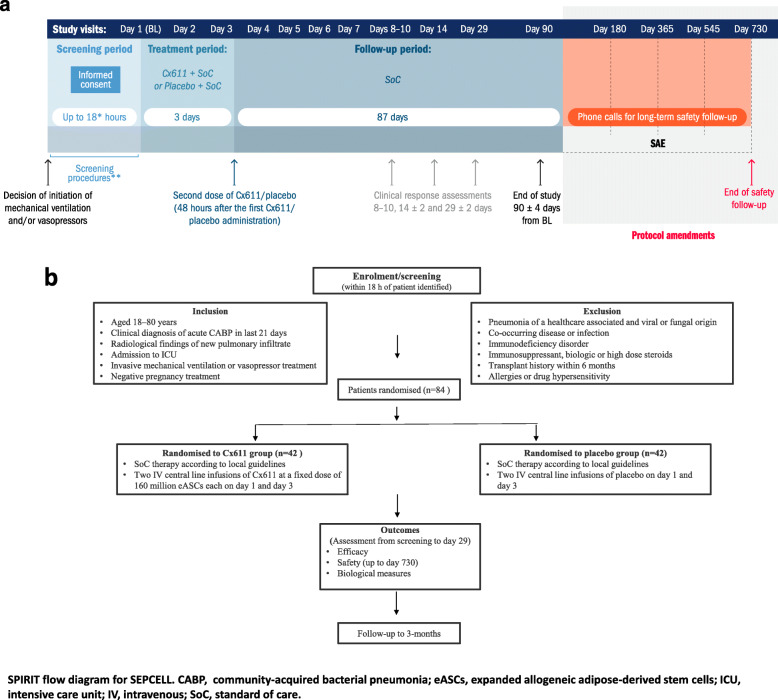
**a** Study design for SEPCELL. A phase Ib/IIa, randomised, multicentre, double-blind, placebo-controlled study to assess the safety, tolerability and efficacy of eASCs (Cx611) administered intravenously, in addition to SoC therapy, to patients with sCABP. An amendment to the study protocol extended the follow-up period to 2 years (Table 2). **b** Trial flow diagram for SEPCELL. *BL* baseline, *CABP* community-acquired bacterial pneumonia, *SPIRIT* Standard Protocol Items: Recommendations for Interventional Trials, *eASC* expanded allogeneic adipose-derived mesenchymal stem cell, *IV* intravenous, *SAE* serious adverse event, *sCABP* severe community-acquired bacterial pneumonia, *SoC* standard of care. *From initiation of mechanical ventilation and/or vasopressors administration; **Screening procedures will start as soon as informed consent is signed

This protocol (version 1, July 2015) follows guidance from the Standard Protocol Items: Recommendations for Interventional Trials (SPIRIT) [30]. A SPIRIT schedule of enrolment, interventions and assessment is provided in Fig. 2. SEPCELL is a phase Ib/IIa, randomised, double-blind, parallel group, placebo-controlled, multicentre trial that planned to enrol 180 patients with sCABP. The study will be conducted from January 2017 to December 2021 in Belgium, France, Lithuania and Spain in over 20 centres. Once eligibility is confirmed, subjects treated in an ICU for sCABP will receive standard-of-care (SoC) according to local guidelines plus two 80 mL intravenous (IV) central line infusions of Cx611 at a fixed dose of 160 million cells (240 mL/hr), or placebo (two 80 mL IV central line infusions [240 mL/hr] of Ringer lactate solution). The randomisation and the first infusion of Cx611 or placebo will be performed as early as possible within the first 18 h of patients fulfilling at least one of the two major criteria of severity for CAP (i.e. from the initiation of invasive mechanical ventilation or vasopressors) (Fig. 3a, b). The day of administration of the first dose of Cx611 (or placebo) will be considered day 1 of the study. The maximum screening duration will be 18 h and treatment duration will be 3 days (Fig. 3a, b). The study will permit concomitant SoC, including antibiotic and other therapies in the ICU, in an add-on design. Patient stratification will be considered based on CABP severity criteria at inclusion: shock requiring vasopressors or invasive mechanical ventilation, or both.

### Study population

The reference population will consist of adult patients admitted to the ICU with sCABP. Eligible patients will include those with clinical diagnosis of acute CABP (developed within ≤21 days past).

### Patients and public involvement

Although patients were not involved in the conception or design of the study, the study concept was approved by a public grant from the EU Horizon Grant commission.

### Participant eligibility

#### Inclusion criteria

Inclusion criteria consist of: age between 18 and 80 years; body weight between 50 and 100 kg; clinical diagnosis of acute CABP (developed within ≤21 past days) based upon the presence of two relevant symptoms (fever, tachypnoea, leukocytosis or hypoxaemia) and radiographic findings of new pulmonary infiltrate(s); having sufficiently severe pneumonia necessitating ICU management, and requiring either invasive mechanical ventilation or treatment with vasopressors for at least 2 h; female subjects must either have no childbearing potential or have negative serum or urine pregnancy test; sexually active subjects (of both sexes) must agree to use contraception for the entire duration of the study, or for 3 months after the last dose of the investigational medicinal product, whichever occurs later; signed informed consent provided by the subject (or relatives or legal representative). The informed consent form includes information that data will be recorded, collected, processed and may be transferred to European Economic Area (EEA) or non-EEA countries in accordance with the European Union Data Protection Regulation (2016/679).

#### Exclusion criteria

Exclusion criteria consist of: hospital-acquired, healthcare-associated or ventilator-associated pneumonia; pneumonia of exclusively viral or fungal origin; known or suspected *Pneumocystis jirovecii* pneumonia; aspiration pneumonia; known active tuberculosis; a history of post-obstructive pneumonia; cystic fibrosis; any chronic lung disease requiring oxygen therapy at home; infection in another organ caused by the same pathogen; expectation for development of a rapidly fatal disease within 72 h after randomisation; inability to maintain a mean arterial pressure ≥ 50 mmHg prior to screening despite utilisation of vasopressors and IV fluids; not being expected to survive for 3 months due to other medical conditions or diseases; subjects with primary or metastatic lung cancer or with anticipated chemotherapy within the next 90 days; haematopoietic and lymphoreticular malignancies, unless in remission; a known primary immunodeficiency disorder or acquired immune deficiency syndrome with CD4 count < 200 cells/mm^3^ or not receiving highly active antiretroviral therapy; receiving immunosuppressant therapy or chronic high doses of steroids; granulocytopoenia (not due to sepsis); receiving any stem cell, organ or bone marrow transplant within the past 6 months; undergoing treatment with a biological agent, or plasma exchange treatment within the last 8 weeks; receiving or having received another investigational medication within 90 days prior to the start of the study; allergies or hypersensitivity to antibiotics and/or any component of CryoStor CS10®; a known liver function deficiency; hospitalisation in the preceding 15 days; conditions resulting in a New York Heart Association Class IV functional status; end-stage neuromuscular disorders that impair weaning; subjects with complete quadriplegia.

### Interventions

Subjects will receive two 80 mL central line infusions (240 mL/hr. over 20–30 min) of IV allogeneic Cx611, at a fixed dose of 160 million cells on day 1 and day 3, respectively; or placebo. Placebo (Ringer Lactate) will be given also through a 20–30 min (240 mL/hr) IV central line infusion at the same quantity (total volume of 80 mL) and following the same schedule than the active treatment.

### Follow-up

All patients will be followed up for a period of at least 3 months.

### Withdrawal

In accordance with the Declaration of Helsinki, patients, or upon their legally authorised representatives’ decision, will be free to withdraw from the study at any time if they wish to, for any reason specified or unspecified. Withdrawal from the study will not affect or detriment the patient’s further care or treatment. Patients have the right to withdraw their consent at any time, partially or totally; however, all data collected until the time of withdrawal will be used in the analyses. Potential criteria for discontinuing or modifying allocated interventions include: occurrence of adverse events that justify the withdrawal from the study; the decision of a subject to withdraw from the study; major protocol deviations; pregnancy.

### Outcome measures

The safety endpoints assessed throughout the study will include incidence of treatment-emergent AEs, including changes in vital signs and signs of allergic reactions such as anaphylaxis (Table 1). Changes in 12-lead electrocardiograms (ECGs) will be assessed from screening, day 1 and day 3 (both post-dose). All AEs will be judged as being related or not to the study treatment.

**Table 1:** Summary of data collection for SEPCELL study [38, 39]

Data collection			
Demographic and baseline data	Safety data	Efficacy data	Biological data
• Date of birth/age • Gender • Race • Medical history and prior medication taken within 2 weeks before the inclusion in the study • All patients will undergo a complete physical examination at screening and day 1 pre-dose	• All AEs, including TEAEs • Physical examination • Signs of allergic reactions • Vital signs • 12-lead ECG • Laboratory safety assessments • Anti-HLA/donor antibodies	• Ventilator-free days • Vasopressor treatment-free days • Ventilator and vasopressor treatment-free days • Clinical response • APACHE II score • SOFA score	• Anti-HLA/donor antibodies • T-cell response • RNA expression profile of leukocytes and protein levels of biomarkers

*AE* adverse event, *APACHE* Acute Physiology A-and Chronic Health Evaluation, *ECG* electrocardiogram, *RNA* ribonucleic acid, *SOFA* Sepsis-related Organ Failure Assessment, *TEAE* treatment-emergent adverse event

Efficacy endpoints measured will include mechanical ventilator and vasopressor treatment-free days over 28 days. Subjects’ clinical response will be assessed during visits on days 8–10, 14 and 29. Response will be determined as cure, non-response or indeterminate. Indications of non-response related to pneumonia will include persistence/progression of baseline signs/symptoms; indications of non-response unrelated to pneumonia will include any other cause of clinical response failure that in the investigator’s judgement is unrelated to the index pneumonia (e.g. myocardial infarction, pulmonary thromboembolism). Time to clinical cure, duration of antibiotic treatment, rate of pneumonia recurrence/re-infection after clinical cure, and time to recurrence/re-infection of pneumonia after clinical cure at clinical response assessment will be recorded.

### Participant timeline

Once a suitable patient has been identified and following informed consent, the maximum screening duration will be 18 h (Fig. 3a). However, initiation of treatment with Cx611/placebo should be performed as early as possible within this 18 h window. The treatment period (day 1 and day 3) starts with the administration of the first Cx611/placebo dose and ends with administration of the second dose. Premature discontinuation can occur if the patient withdraws from the study before day 90 and will lead to an early termination visit.

### Sample size

The results from this study will be exploratory in nature; hence, there is no hypothesis testing comparing outcomes between treatment arms. As little is presently known about the outcome measures used in the study of stem cells, SEPCELL will provide the initial dataset necessary for determining endpoints and enable preliminary estimates of effect size for the design of future efficacy-finding studies of Cx611 used as add-on therapy in patients with sCABP requiring mechanical ventilation and/or vasopressor administration.

### Recruitment

Since patients with sCABP require prompt treatment, study enrolment will take place at each study site during a short time window (up to 18 h), starting from when the patient is first identified to fulfil the appropriate severity criteria and ending with the administration of the first dose of study treatment. Each participating centre will keep a log of all screened patients with sCABP, and investigators will be requested to record the reason(s) for not including patients who were screened but not enrolled.

### Randomisation and allocation

After performing the screening visit and verifying inclusion and exclusion criteria and subject’s eligibility, investigators will place a screening and approval call to the clinical coordinating centre (CCC) physicians. The CCC will provide a randomisation authorisation number, which will allow the patient to be randomised within the study. Patients will then be randomised in a 1:1 ratio to receive Cx611 or placebo by interactive response technology (IRT). The IRT will assign a randomisation number to the patient, which will be used to link the patient to a treatment arm and will specify a unique medication number for the package of the investigational medicinal product (a pack containing two vials of Cx611 or placebo properly labelled) to be dispensed to the patient at each administration date. The IRT will produce a patient randomisation list via a validated system that automates the random assignment of patient numbers to randomisation numbers. A separate medication list will be produced via the IRT provider also using a validated, automated method to randomise the assignment of medication numbers to packs containing the investigational medicinal product.

### Blinding

Both patients and investigators (or designated personnel) who collect the AE data and evaluate the clinical outcomes of sCABP will be blinded to treatments. Treatments will be prepared by unblinded personnel for administration in a blinded location, and this individual(s) will not be allowed to participate in any efficacy assessment of the sCABP during the study. The unblinded personnel will not be permitted to share information about the treatment with any member of the blinded team. To ensure double blinding, the primary packaging of Cx611 and placebo will be identical. Additionally, a specific blinding plan at each site will document all personnel involved in the trial and their responsibilities.

### Data management

Data management and handling will be conducted according to the study-specific Data Management Plan in accordance with International Conference on Harmonization guidelines [31] and clinical research organisation standard operating procedures (SOPs). Data entry, validation and data queries will be handled by the Trial Form Support (TFS) data management team. Data will be subjected to validation according to TFS SOPs in order to ensure accuracy in the collected case report form data. Before database closure, reconciliation will be performed between the SAEs entered in the safety database and the study database. Any deviations, i.e., discrepancies and additions from the process defined in the Data Management Plan, will be described in a study-specific data management report. When data for the primary endpoint are available and before database lock, a blinded adjudication committee will review subject evaluability, sCABP clinical response assessment and patient assignment processes.

### General statistical methods

In general, data will be reported by means of summary statistics. Continuous data will be presented with the number of observations, mean value, standard deviation, and minimum, median and maximum value. Categorical data will be presented as counts and percentages when applicable. Individual patient data will be listed. The results of all laboratory test results, physical examination findings, ECGs and vital signs will be presented in data listings; safety laboratory data will be presented by absolute and changes from baseline values by visit. All abnormalities will be assessed for potential clinical relevance.

### Protocol amendments

Protocol amendments are summarised in Table 2.

**Table 2:** Summary of protocol amendments to SEPCELL study

Protocol amendment number [date]	Rationale for amendments
**1** [17/11/2016]	1. **Study objectives updated to incorporate an additional secondary objective to explore longer-term safety:** Follow-up safety (only SAEs) at months 6 and 12 after the first IMP dose administration (day 1) 2. **Study objectives updated to incorporate an exploratory objective to explore longer-term safety:** Safety data collection (SAEs collection via phone call) at months 18 and 24 These changes were made to meet the requests received from the Spanish regulatory agency (AEMPS) where the clinical trial application was submitted and approved.
**2** [01/08/2017]	1. **Clinical study protocol updated so CryoStor® CS10, an excipient of Cx611, was also used in the placebo arm:** DMSO in CryoStor® CS10 is chemically changed by the human body and subsequently secreted, causing a distinct garlic odor to be exhaled approximately 48 h after administration. To reduce the chances of accidental unblinding, CryoStor® CS10 was added to the placebo arm. New placebo kits provided to study sites included CryoStor® CS10 and Ringer lactate solution 2. **ICFs for subject, legal representative and independent physicians were also updated to highlight changes made to the placebo arm**
**3** [28/05/2018]	1. **Contraception language amended to follow the Clinical Trial Facilitation Group 2014 recommendations related to contraception and pregnancy testing in clinical trials** 2. **Exclusion criteria updated to exclude patients with a history of malignancy within 5 years before enrolment** 3. **ICF updated to incorporate updated European data privacy laws (General Data Protection Regulation)** These changes were made to meet requirements and implement feedback received from European regulatory agencies/ethics committees where the clinical trial application was submitted and approved.
**4** [04/09/2019]	1. **Safety reporting contact details and process were updated** 2. **Information regarding IMP shelf life was updated to reflect product re-test period had been extended** (previous re-test period of 18 months if stored at − 140 °C or below) and updated expiry dates were reflected in the IRT
**5** [19/12/2019]	1. **Early closure of study enrolment** Due to persistent recruitment challenges throughout the study and to avoid not meeting study enrolment goals in a sufficient period of time, enrolment will be closed early^a^ 2. **Secondary objective was updated to an exploratory objective** (*understand the MoA of Cx611 in patients with sCABP by identifying the pro-inflammatory and anti-inflammatory pathways through which Cx611 may affect the underlying processes of sepsis*) Due to reduced number of subjects enrolled in the study, data analysis will no longer be powered to detect statistical difference, but they will provide useful information on trends. Also tests that are no longer of interest will be removed to allow flexibility to test for specific biomarkers of most interest nearer the time of sample analysis 3. **Study unblinding will occur after day 90 data has been collected and analysed for all subjects** To allow the investigators to be unblinded earlier, efficacy assessments post-day 90 will be removed (previously at day 180 and day 365). Safety assessments at day 180, day 365, month 18 and month 24 will remain 4. **Safety and efficacy data will be summarised via descriptive statistics only** Due to early closure of enrolment, the total number of enrolled subjects will be too low to detect any safety and efficacy signals, and, therefore, any statistical inference including 95% CIs and *p* values may be misleading

*AEMPS* Agencia Española de Medicamentos y Productos Sanitarios, *CI* confidence interval, *DMSO* dimethyl sulfoxide, *eASC* expanded adipose derived mesenchymal stem cells, *ICF* informed consent form, *IMP* investigational medical product, *IRT* interactive response technology, *MoA* mode of action, *SAE* serious adverse event, *sCABP* severe community-acquired bacterial pneumonia

^a^As of July 2020, the trial is active but no longer enrolling participants

### Confidentiality

All study-related information and participant information will be stored securely at the study site in areas with limited access. Password-protected access systems will be used to ensure the confidentiality of local databases.

### Publications

The list of authors of the publication will be defined according to International Committee of Medical Journal Editors criteria, involvement in trial design, oversight, number of evaluable patients enrolled, analysis and interpretation of data, and preparation of manuscript. The study will only be published once it has been finished and the final analysis is completed; the final manuscript must be approved by all the authors before publication [32]. Medical writing support will be utilised as required.

### Ethics and dissemination

If the patient is unable to comprehend the scope of the trial prior to enrolment due to altered mental status associated with the underlying pneumonia (or any other disease), written informed consent to participate in the study must be obtained from the patient’s legally acceptable representative, as required by national laws, respective regulations and institutional review boards/independent ethics committees/regional ethics boards. Written informed consent will be sought from the patient as soon as he/she becomes capable of comprehending the scope of the trial. The study results (publications, conference presentations) will be published in peer-reviewed, open access journals and conferences.

## Discussion

SEPCELL is the first trial to assess the effects of eASCs in sCABP. Due to the associated complications and high mortality rate of sCABP, treatment remains a key challenge and a global public health issue [1]. With current therapies ineffective for many patients [3], there is an unmet need for novel therapies to address these challenges.

In animal models, the immunomodulatory properties of MSCs were found to significantly reduce mortality via a combination of reduced inflammation, production of anti-microbial effectors and increased phagocytosis [11, 33]. To date, there are no large-scale clinical trials demonstrating efficacy of stem cell therapy in sCABP. However, results from a phase I dose-escalation clinical trial of MSCs in septic shock provided data suggesting MSCs in doses up to 250 million cells are to be deemed safe and well-tolerated (NCT02421484 [34];). A phase II trial to assess the efficacy and safety of MSCs in the treatment of septic shock is now planned (NCT03369275). Furthermore, results from a phase I and phase IIa clinical trial (NCT01775774; NCT02097641) have shown stem cell therapy to be well tolerated in patients with acute respiratory distress syndrome (ARDS) [35, 36], with two phase II trials currently ongoing in ARDS in the US and the Republic of Korea (NCT03818854, STAT; NCT02112500, STELLAR, respectively).

The current trial will help advance our knowledge of the anti-inflammatory effects of eASCs, such as effects on T-cell response, RNA expression profiles of blood leukocytes and plasma concentration of biomarkers in this patient population, building on the promising results provided by preclinical and phase I data for Cx611 [19–21]. SEPCELL will also provide insights on the efficacy of Cx611, including its impact on the need for use of mechanical ventilation and/or vasopressors, time to clinical cure and rate of pneumonia recurrence/re-infection after clinical cure in patients with sCABP. Although mortality will not be assessed during the SEPCELL study, data on the safety and tolerability of eASCs in patients with sCABP will provide key information to facilitate the design of subsequent clinical trials.

To conclude, SEPCELL is a multicentre, double blind, randomised controlled trial assessing the safety, tolerability and efficacy of eASCs (Cx611) as adjunctive therapy for patients with sCABP. The novel MoA offered by eASCs may address the underlying immune dysregulation caused by sCABP, and, thereby, offer another treatment option for patients with this disease and ultimately reduce patient mortality [37]. In addition to advancing our understanding of the MoA of Cx611, data from this completed trial will provide evidence on the safety, tolerability and efficacy of Cx611 in patients with sCABP. Analysis of study results and outcomes will be critical for the design of further confirmatory clinical investigations in terms of definition of endpoints, key biomarkers of interest and sample size determination.

## Supplementary Information

**Additional file 1.** SPIRIT checklist.

**Additional file 2.** World Health Organization Trial Registration Data Set is available.

## Data Availability

The datasets, including the redacted study protocol, redacted statistical analysis plan, and individual participants data supporting the results of the study, will be made available after the publication of study results within 3 months from initial request, to researchers who provide a methodologically sound proposal. The data will be provided after its de-identification, in compliance with applicable privacy laws, data protection and requirements for consent and anonymization*.* A completed SPIRIT checklist is available in Additional file 1; the World Health Organization Trial Registration Data Set is available in Additional file 2.

## Abbreviations

AE: Adverse event
ARDS: Acute respiratory distress syndrome
ASC: Adipose-derived mesenchymal stem cell
BM-MSC: Bone-marrow-derived mesenchymal stem cell
CABP: Community-acquired bacterial pneumonia
CAP: Community-acquired pneumonia
CCC: Clinical coordinating centre
eASC: Expanded ASC
ECG: Electrocardiograms
IRT: Interactive response technology
MoA: Mode of action
MSC: Mesenchymal stem cell
SAE: Serious adverse event
sCABP: Severe CABP
SoC: Standard of care
SOP: Standard operating procedure
