# Supplementary material for: A phase Ib/IIa, randomised, double-blind, multicentre trial to assess the safety and efficacy of expanded Cx611 allogeneic adipose-derived stem cells (eASCs) for the treatment of patients with community-acquired bacterial pneumonia admitted to the intensive care unit
Source: BMC Pulm Med. 2020 Nov 25;20:309. doi: 10.1186/s12890-020-01324-2 (PMC7686829; doi:10.1186/s12890-020-01324-2)
Supplement: Supplementary file 2 — Additional file 2. World Health Organization Trial Registration Data Set is available. [file 12890_2020_1324_MOESM2_ESM.docx]

| **Data Category** | **Information** |
| --- | --- |
| Primary Registry and Trial Identifying Number: | NCT03158727 |
| Date of Registration in Primary Registry | May 9, 2017 |
| Secondary Identifying Numbers | EudraCT No.: 2015-002994-39 |
| Source(s) of Monetary or Material Support | This study is funded by the European Union’s Horizon 2020 Research and Innovation Program under grant agreement number 681031. The study sponsor is TiGenix SAU (a wholly-owned subsidiary of Takeda Pharmaceutical Company Limited). |
| Primary Sponsor | TIGENIX, S.A.U.  C/ Marconi, 1 Parque Tecnológico de Madrid  28760, Tres Cantos, Madrid |
| Secondary Sponsor(s) | - European Commission - Centre Hospital Regional Universitaire de Limoges - Cliniques universitaires Saint-Luc- Université Catholique de Louvain - Academisch Medisch Centrum - Universiteit van Amsterdam (AMC-UvA) - Hospital San Carlos, Madrid |
| Contact for Public Queries | Name: Dr. Pierre-François Laterre  E-mail: [pierre-francois.laterre@uclouvain.be](mailto:pierre-francois.laterre@uclouvain.be) |
| Contact for Scientific Queries | Name: Dr. Pierre-François Laterre  Address: Intensive Care Unit, St Luc University Hospital, 10 Avenue Hippocrate, 1200 Brussels, Belgium.  E-mail: [pierre-francois.laterre@uclouvain.be](mailto:pierre-francois.laterre@uclouvain.be) |
| Public Title | A phase Ib/IIa, randomised, double-blind, multicentre trial to assess the safety and efficacy of expanded Cx611 allogeneic adipose-derived stem cells (eASCs) for the treatment of patients with community-acquired bacterial pneumonia admitted to the intensive care unit |
| Scientific Title | A phase Ib/IIa, randomised, double blind, parallel group, placebo controlled, multicentre study to assess the safety and efficacy of expanded Cx611 allogeneic adipose-derived stem cells (eASCs) for the intravenous treatment of adult patients with severe community-acquired bacterial pneumonia (sCABP) and admitted to the intensive care unit |
| Countries of Recruitment | Belgium, France, Spain. |
| Health Condition(s) or Problem(s) Studied | Community-acquired bacterial pneumonia. |
| Intervention(s) | - Experimental: Cx611   Subjects treated in an intensive care unit for sCABP, but that may be screened at the emergency department, will receive SoC therapy according to local guidelines plus two intravenous central line infusions of Cx611 at a fixed dose of 160 million expanded allogeneic adipose-derived stem cells (eASCs) each.   - Intervention: Placebo Comparator   Subjects treated in an intensive care unit for sCABP, but that may be screened at the emergency department, will receive SoC therapy according to local guidelines plus two intravenous central line infusions of Ringer’s Lactate.  Intervention: Other: Placebo. |
| Key Inclusion and Exclusion Criteria | Inclusion criteria include: Adult subjects of either gender (aged ≥18 years and ≤80 years old). Clinical diagnosis of acute community acquired bacterial pneumonia based on the presence of two relevant signs (fever, tachypnoea, leukocytosis, or hypoxemia) and radiographic findings of new pulmonary infiltrate/s. Subjects with pneumonia of sufficient severity requiring ICU management. Non-pregnant females. Signed informed consent or the designated relative or legal representative according to local guidelines.  Exclusion criteria include: Subjects with pneumonia from a non-community acquired origin. Subjects with any co-occurring diseases, pathogenic infections, or immunodeficiency disorders or history of malignancy in the 5 years before treatment. Subjects who received medical treatments in the previous 8 weeks including biologic agents, immunotherapy plasma exchange and/or stem cell therapy. Subjects with a history of recent hospitalisation. Subjects with known allergies, drug hypersensitivities or liver function impairment. Inability to maintain a sufficient mean arterial pressure despite the presence of vasopressors and intravenous fluids. |
| Study Type | Interventional  Allocation: Randomized  Intervention Model: Parallel Assignment  Masking: Quadruple (Participant, Care Provider, Investigator, Outcomes Assessor)  Primary Purpose: Safety and efficacy of treatment  Phase Ia/IIb |
| Date of First Enrolment | January 30, 2017 |
| Sample Size | Target: 180  Actual: 84 |
| Recruitment Status | Active, not recruiting |
| Primary Outcome(s) | Safety profile of 2 allogeneic Cx611 central line infusions at a dose of 160 million cells each. |
| Key Secondary Outcomes | Clinical efficacy of Cx611 in terms of a reduction of the duration of mechanical ventilation and/or vasopressors needed and/or improved survival, and/or clinical cure of the sCABP, and other efficacy-related endpoints. |
| Ethics Review | The study was approved by competent authorities and ethics committees in Belgium, Spain, Lithuania, Italy, Norway and France. |
| Completion date | December 16 2021 (estimated) |
| Summary Results | No results posted. |
| IPD sharing statement | \| Plan to Share IPD: \| Yes \| \| --- \| --- \| \| Plan Description (as of 22.09.2020): \| Takeda makes patient-level, de-identified data sets and associated documents available for all interventional studies after applicable marketing approvals and commercial availability have been received (or program is completely terminated), an opportunity for the primary publication of the research and final report development has been allowed, and other criteria have been met as set forth in Takeda's Data Sharing Policy (www.TakedaClinicalTrials.com). To obtain access, researchers must submit a legitimate academic research proposal for adjudication by an independent review panel, who will review the scientific merit of the research and the requestor's qualifications and conflict of interest that can result in potential bias. Once approved, qualified researchers who sign a data sharing agreement are provided access to these data in a secure research environment. \| |
